# The Effect of Head Orientation on Perceived Gaze Direction: Revisiting Gibson and Pick (1963) and Cline (1967)

**DOI:** 10.3389/fpsyg.2016.01191

**Published:** 2016-08-10

**Authors:** Pieter Moors, Karl Verfaillie, Thalia Daems, Iwona Pomianowska, Filip Germeys

**Affiliations:** ^1^Laboratory of Experimental Psychology, Department of Brain and Cognition, KU LeuvenLeuven, Belgium; ^2^The Leon Schiller National Higher School of Film, Television and TheatreLodz, Poland; ^3^Department of Work and Organisation Studies, KU LeuvenLeuven, Belgium

**Keywords:** gaze perception, perceived gaze direction, overshoot effect, towing effect, joint attention

## Abstract

Two biases in perceived gaze direction have been observed when eye and head orientation are not aligned. An overshoot effect indicates that perceived gaze direction is shifted away from head orientation (i.e., a repulsive effect), whereas a towing effect indicates that perceived gaze direction falls in between head and eye orientation (i.e., an attraction effect). In the 60s, three influential papers have been published with respect to the effect of head orientation on perceived gaze direction (Gibson and Pick, 1963; Cline, 1967; Anstis et al., 1969). Throughout the years, the results of two of these (Gibson and Pick, 1963; Cline, 1967) have been interpreted differently by a number of authors. In this paper, we critically discuss potential sources of confusion that have led to differential interpretations of both studies. At first sight, the results of Cline (1967), despite having been a major topic of discussion, unambiguously seem to indicate a towing effect whereas Gibson and Pick’s (1963) results seem to be the most ambiguous, although they have never been questioned in the literature. To shed further light on this apparent inconsistency, we repeated the critical experiments reported in both studies. Our results indicate an overshoot effect in both studies.

## Introduction

**FIGURE 1:**
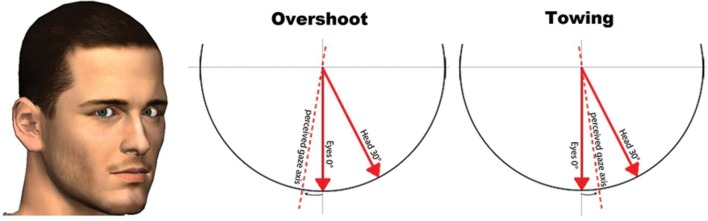
**An illustration of the overshoot **(left)** and towing effect **(right)****.

The ability to accurately determine where another person is looking is a crucial function in everyday interaction and communication (Argyle and Cook, 1976; Emery, 2000). Generally, observers are quite accurate in determining the direction of another person’s gaze (e.g., Gibson and Pick, 1963; Symons et al., 2004; Bock et al., 2008). Nevertheless, some consistent biases in perceived gaze direction have also been reported, mainly with respect to the integration of eye and head orientation. In the 60s, three pioneering studies have been conducted that documented these biases for the first time (Gibson and Pick, 1963; Cline, 1967; Anstis et al., 1969). For example, Gibson and Pick (1963) studied the influence of head orientation on perceived gaze direction by instructing participants to indicate whether or not a looker was looking at him/her (i.e., a dyadic gaze task). When the head was oriented straight to the participant, participants were very accurate. A head turn of 30° either to the left or to the right of straight ahead revealed a systematic error. According to Anstis et al. (1969), these results indicated that perceived gaze direction shifted opposite to the direction of the head. The highest frequency of responses that the looker was looking at the participant was given when the looker (with her head turned to the participant’s right^1^) was actually gazing at the participant’s right ear (which was ∼2.9° of visual angle from the center of the participants’ face). This head turn effect has been dubbed the *overshoot effect* by Langton et al. (2000) since estimated gaze location is biased in a direction opposite to the orientation of the head (**Figure 1**, middle panel).

Subsequent studies of Cline (1967) (Experiment IIB) and Anstis et al. (1969) relied on a triadic gaze task in which observers have to indicate at which point in space they perceive another person to be looking. Both studies replicated and extended the findings of Gibson and Pick (1963). Anstis et al. (1969) reported an overshoot effect when eye gaze had to be estimated when the head of a looker was turned. Cline’s (1967) results of Experiment IIB have been debated, however, (Anstis et al., 1969; Vine, 1971; von Cranach and Ellgring, 1973; Kluttz et al., 2009).

In apparent contradiction with an overshoot effect, Maruyama and Endo (1983) reported a *towing effect* in which head and eye information are integrated in such a way that perceived gaze is pulled toward the position of the head and thus falls somewhere in between the orientation of the head and eyes (**Figure 1**, right panel). In their experiment, Maruyama and Endo (1983) independently manipulated eye and head orientation and concluded that perceived gaze direction always falls somewhere in between both orientations.

In sum, with respect to the influence of head orientation on perceived gaze direction, both an overshoot and a towing effect have been observed throughout the literature (Gibson and Pick, 1963; Cline, 1967; Anstis et al., 1969; Maruyama and Endo, 1983; Maruyama et al., 1985; Langton et al., 2000, 2004; Seyama and Nagayama, 2005; Todorović, 2006; Ricciardelli and Driver, 2008; Kluttz et al., 2009).

Upon reviewing the literature, we checked all papers citing either Gibson and Pick (1963) or Cline (1967) (cited 336 and 167 times, respectively, according to Google Scholar on June 9, 2016). From these papers, we selected only those in which the direction of the effect of head orientation on perceived gaze direction was explicitly interpreted (**Supplementary Table S1**). The results indicated that 12 out of 22 studies citing Cline (1967) took his results as evidence for a towing effect and 10 out of 22 as evidence for overshoot effect. Four out of 25 studies citing Gibson and Pick (1963) took their results as evidence for towing effect and 21 out of 25 took their results as evidence for overshoot effect^2^.

Given this discrepancy in the literature, the goal of the present study is twofold. We will first discuss for both studies how one potentially can arrive at different interpretations of the same result. Second, in order to shed light on the replicability of this discrepancy, we repeated both experiments in order to assess in which direction perceived gaze would be biased (i.e., overshoot or towing) for both experimental set-ups.

### Differing Interpretations for Gibson and Pick (1963) and Cline (1967) – Experiment IIB

For the sake of brevity, here we present a summary of a detailed discussion of both findings. For an extensive version of this discussion, the reader is referred to the Supplementary Materials.

To reiterate, in their study Gibson and Pick (1963) assessed the influence of head orientation on perceived gaze direction by instructing participants to indicate whether a looker (who was instructed to look at various targets placed behind the participant) was looking at him/her or not (also known as a dyadic gaze task). The authors observed that perceived gaze was indeed shifted in function of head orientation as revealed by the fact that, for the head oriented 30° to the left or the right, the target number associated with the highest frequency of “looking at me” responses shifted. Essentially, the main argument as to why the results can not be unambiguously interpreted is that the authors never explicitly define whether head orientation (i.e., left or right) is defined from the lookers’ or the participants’ viewpoint. As outlined in the detailed discussion, this yields several of their descriptions of their results ambiguous with respect to the direction of the observed effect.

The case of the report of Cline (1967) is more complex, however, which is also highlighted by the amount of studies that have interpreted the results either way. In his study, Cline wished to assess the accuracy of perceived gaze direction in a triadic gaze task. That is, participants had to indicate at which point on a target board a looker was perceived to be looking. The main points of discussion of the results reported in Cline’s study are the results of Experiment IIB. Here, perceived gaze direction was measured in two different head orientation conditions (0° and 30° rotated to the right) for various targets located on the horizontal midline of a target board. Because Cline also never defines whether head orientation should be interpreted from the lookers’ or the participants’ viewpoint, it has frequently been argued that the results of Cline are to be interpreted as an overshoot effect either (1) because he interprets the results himself as being in line with Gibson and Pick (1963) or (2) because he mixed up the target labels in the table in which the experimental results are depicted. In this interpretation, one particular aspect of Cline’s experimental set-up has been overlooked, however. That is, all participants had to judge gaze direction of the looker through a *half-silvered mirror*. Due to this, when viewing the looker through a mirror, the participant will always perceive the head orientation of the looker in the same direction as the looker rotates his/her head and therefore, the reference frame for left or right is no longer important. Indeed, a head orientation 30° to the right is perceived as such by the participant. Hence, we argue that all results reported by Cline (1967) more than likely can be interpreted as a towing effect.

In sum, we argue that although the study of Gibson and Pick (1963) is generally agreed upon as reporting an overshoot effect, it actually is the most ambiguous study. That is, because the authors never report whether head orientation should be interpreted from the looker’s or participant’s viewpoint, the direction of the effect can not be interpreted unambiguously. In contrast, experiment IIB reported in Cline (1967), the results of which have been a major topic of discussion, very likely indicated a towing effect.

In the remainder of the paper, we will report on two replication experiments we conducted. That is, we repeated the experiment reported in Gibson and Pick (1963) as well as Experiment IIB of Cline (1967). In a last section, we will discuss how our findings compare to those reported in the original studies.

## Replication of Gibson and Pick (1963)

In this replication experiment, participants had to indicate whether they perceived a looker to be looking at them or not (i.e., a dyadic gaze task). We manipulated head orientation (0°, 30° to the left, and 30° to the right) and measured “looking at me” responses for a range of targets located behind the participant.

### Materials and Methods

#### Participants and Models

Twelve participants (seven female, five male, age range: 21–38 years) participated in the experiment in return for a monetary reward of 10 euros. All participants were naïve with respect to the purposes of the experiment and had normal or corrected-to-normal vision. The study was approved by the local ethics committee, and every participant signed an informed consent at the start of each experiment, conform to the ethical standards laid down in the 1964 Declaration of Helsinki. Because the original study used lookers rather than computer-generated images, and because this increases the ecological validity of the study (Lassalle and Itier, 2015), we also relied on lookers. Here, we used two different lookers (for six participants each) to assess the consistency of a potential effect of head orientation across different lookers (one 32 years old female looker, one 41 year old male looker). The experiment lasted approximately 60 min.

#### Apparatus and Stimuli

**FIGURE 2:**
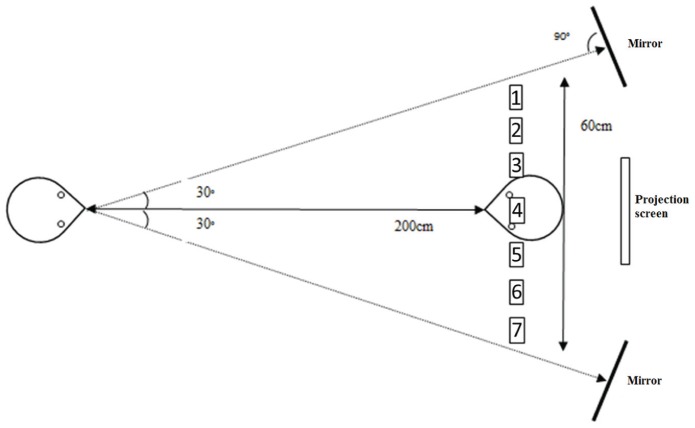
**A schematic illustration of the experimental setup for the Gibson and Pick (1963) replication experiment.** The looker **(left)** and participant **(right)** were facing each other at a distance of 200 cm. The targets were located on a music stand behind the participant. The projection screen was placed behind the participant on a chair standing on a table to make it clearly visible to the looker. The mirrors were located at exactly 30° to the left and right of the looker. Note that, for illustration purposes, the relations between targets and mirrors are not proportional to the actual set-up.

Our experimental set-up is depicted in **Figure 2.** We aimed at mirroring the set-up as reported by Gibson and Pick (1963) as closely as possible. The looker and participant were seated in a chair and facing each other. The lookers’ chair could be adjusted in height to be in line with the participants’ eyes. All targets were placed behind the participant and attached horizontally to a music stand. The music stand could be adjusted in height such that target number 4 would always be aligned with the participants’ bridge of the nose. Targets were separated 2.86° of visual angle from the lookers’ point of view (i.e., 2.86°, 5.72°, and 8.58° to the left and right of the participants’ bridge of the nose). Because it was unclear how the looker from the original study achieved reliable head rotation, we used two mirrors that were placed at exactly 30° to either direction. If the looker had to rotate his/her head on a particular trial, he/she had to look straight into the mirror which coincided with that particular head orientation. All experimental conditions were presented to the looker through a computer screen that was placed on a chair on a table above and behind the participant. On each trial, a target number was shown on the screen together with an arrow if the participant had to rotate his/her head on that particular trial. Stimulus presentation and response recording was achieved through custom software written in Python and relying on the PsychoPy software package (Peirce, 2007, 2009).

#### Procedure and Design

On each trial, the participant had to close his/her eyes until the looker indicated that he/she could open them to judge perceived gaze direction. During this period, the looker followed the instructions on the screen to look at the correct target (with an appropriate head orientation if necessary). After the looker indicated that the participant could open his/her eyes, the participant had to indicate whether he/she perceived the looker to be looking at him/her by a response on the keyboard (arrow “up” for “yes” and arrow “down” for “no”) which the participant held on his/her lap. The participant was instructed to judge gaze direction based on the first impression rather than contemplating on it, yet participants were given unlimited response time. After the participants’ response, he/she closed his/her eyes again and the looker initiated the next trial.

We tested all combinations of seven targets and three head orientations 25 times, yielding a total of 525 trials. All conditions were presented in a fully random order. Every block consisted of 105 trials after which participants were encouraged to take a break.

### Results and Discussion

All analyses were performed in R, an open-source statistical programming tool (R Core Team, 2014). All statistical analyses were performed in a Bayesian framework relying on Bayes Factors (BF) calculated using the BayesFactor package (Rouder and Morey, 2012; Rouder et al., 2012; Morey and Rouder, 2015). BFs constitute a *relative* measure of evidence, quantifying how much more likely one statistical model is compared to another. For example, a BF of three for a statistical model including two main effects versus a statistical model including two main effects and their interaction indicates that the data are three times more likely under the former model than the latter. This would indicate that no interaction is present in the data. It should be stressed that a BF does not constitute an absolute measure of model fit, but is always a relative measure of one model compared to another. For clarity, all BFs reported in this study are always relative to the *best fitting model* (i.e., the model that is most likely). Thus, a model for which the BF is 1 indicates the best fitting model (see **Tables 1** and **2**). BFs > 1 then indicate how much more likely the best fitting model is compared to another model. All models for which BFs were computed were ANOVA-style models including random intercepts for participants. In this study, BFs > 3 are considered to be evidence for the best fitting model over the other (Jeffreys, 1961).

**Table 1:** Bayes Factor analysis for Gibson and Pick replication experiment.

Model	Bayes Factor
Head orientation + Looker	1
Head orientation ^∗^ Looker	3.3
Head orientation	3.4
All other models	>100


*Bayes Factors are relative to the best fitting model (the model for which the Bayes Factor is 1). Bayes Factors > 1 thus indicate how much more likely the best fitting model is compared to the other model. A ^∗^ indicates a model containing the main effects and the interaction between the variables.*

**Table 2:** Bayes Factor analysis for the Cline replication experiment.

Model	Bayes Factor
**Looker FG**	
Target + Head orientation	1
Target	1.9
Head orientation	19
All other models	>100
**Looker IP**	
Target ^∗^ Head orientation	1
All other models	>100


*Bayes Factors are relative to the best fitting model (the model for which the Bayes Factor is 1). Bayes Factors > 1 thus indicate how much more likely the best fitting model is compared to the other model. A ^∗^ indicates a model containing the main effects and the interaction between the variables.*

To obtain an estimate of the mean target that yielded the highest frequency of “looking at me” responses, we fitted Gaussian distributions on the obtained frequencies for each head orientation separately. Note that in the case of an overshoot effect, this analysis would yield a shift in the mean target toward lower target numbers for a head oriented to the left and toward higher target numbers for a head orientated to the right of the looker. That is, when the looker rotates his/her head to his/her left and looks at target number 3 and perceived gaze direction is biased in the direction opposite to the head turn, this target would be more likely to receive a higher frequency of “looking at me” responses. An opposite pattern would be predicted in the case of a so-called towing effect.

**FIGURE 3:**
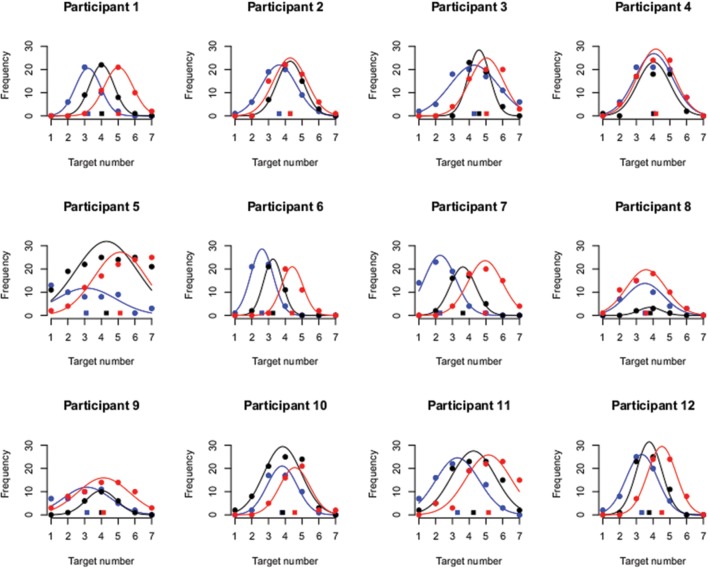
**Individual data.** For each participant, the frequency of “yes, looking at me” responses is plotted (disks) in function of target number for all different looker head orientations (blue = left, black = middle, red = right). The best fitting Gaussian distributions are overlaid with the mean of each distribution plotted at the bottom of each graph (squares).

**FIGURE 4:**
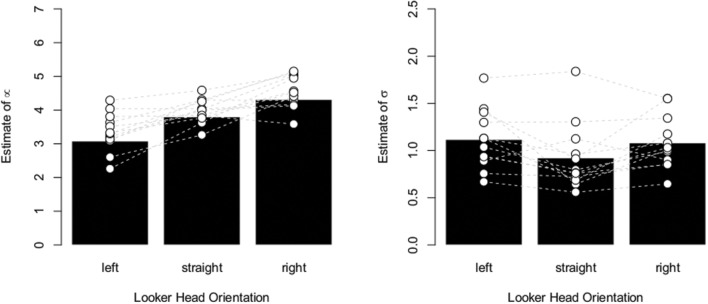
**Aggregate data.** **(Left)** Bar plot depicting the average estimate of the mean of the Gaussian distribution. The estimate increases in function of head orientation in the direction that would be expected in the case of an overshoot effect. The white disks indicate individual data points and the gray dashed line connects data from the same participant. **(Right)** Bar plot depicting the average estimate of the standard deviation of the Gaussian distribution. The data show substantial inter-individual variability and a small tendency of broader distributions for rotated heads is observed.

The results of this analysis are depicted in **Figure 3** for each participant separately. As is apparent, for two–thirds of the participants (1, 3, 5, 6, 7, 9, 11, 12), the data show the ordinal pattern predicted by an overshoot effect (i.e., mean target number for left head orientation < straight head orientation < right head orientation). Furthermore, participants that do not show this ordinal pattern rather show no influence of head orientation on perceived gaze direction rather than a reversal in the direction of a towing effect. This is further substantiated by the average results depicted in **Figure 4.** The BF analysis is summarized in **Table 1.** The best fitting model contains both a main effect of head orientation as well as of the looker. Although the other BFs are close to 3, they pertain only the effects related to the looker, and not to the head orientation. The analysis thus indicates that the data are very consistent with a model including a main effect of head orientation.

Since the effect of head orientation did not interact with the looker, the data in **Figure 4** are also averaged across both lookers. As the Figure indicates, the estimate of the mean of the Gaussian distribution consistently shifted in function of head orientation and in the direction that would be predicted by an overshoot effect. That is, when the head of the looker was oriented 30° to his/her left (and thus to the right of the participant), the average estimate of the mean of the distributions was about 3 (see **Figure 2**). This indicates that this target number (located near the participants’ right ear) yielded the highest frequency of “looking at me” responses, implying that perceived gaze was shifted in the direction opposite to the head orientation of the looker. In contrast with the original study, we also observed small differences between the estimated standard deviations of the Gaussian distributions in function of head orientation. That is, as the head was rotated, participants’ distributions became, on average, slightly broader. However, the evidence for an effect of head orientation on estimated standard deviations was only weak (BF = 2.7).

In sum, we succeeded in replicating the head orientation effect reported in Gibson and Pick (1963). For most participants, the pattern of results was in line with the pattern predicted by an overshoot effect. Thus, although the original study reported by Gibson and Pick (1963) could be interpreted both in terms of an overshoot or towing effect depending on the interpretation of the frame of reference in which head orientation was described, our results give more weight to an overshoot interpretation of the original results.

In the next section, we report on the replication experiment we conducted of Experiment IIB reported in Cline (1967).

## Replication of Cline (1967) – Experiment IIB

The goal of Experiment IIB reported in Cline (1967) was to examine whether head orientation would influence perceived gaze direction in a triadic gaze task. That is, on each trial participants had to indicate at which target a looker was looking with his/her head either straight ahead or oriented 30° to the right. Given the use of a mirror in Cline’s original set-up, we argued earlier that his results more than likely indicated a towing effect.

### Methods

#### Participants

Twelve participants (1 male, 11 female, mean age = 24 years, age range 22 – 33 years) participated in the experiment in exchange for a monetary reward of 10 euros. All participants were naïve with respect to the purposes of the experiment and had normal or corrected-to-normal vision. The study was approved by the local ethics committee, and every participant signed an informed consent at the start of each experiment, conform to the ethical standards laid down in the 1964 Declaration of Helsinki. We used the same two lookers as in the Gibson and Pick (1963) replication experiment. The experiment lasted approximately 45 min.

#### Apparatus and Stimuli

**FIGURE 5:**
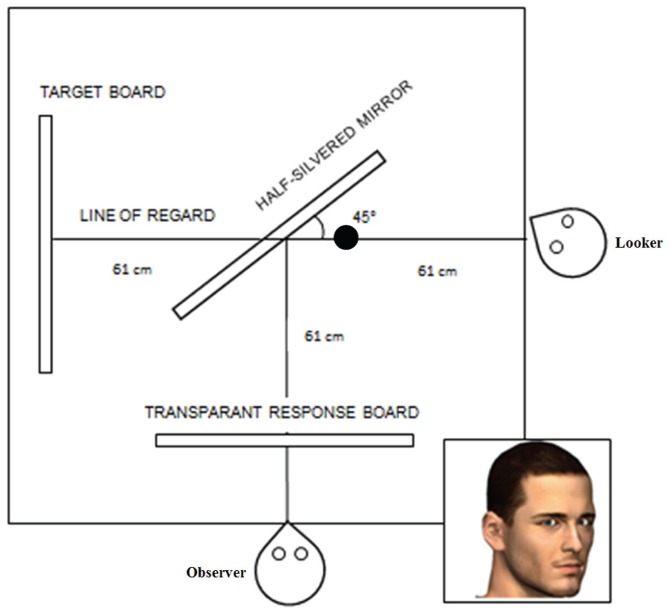
**Experimental set-up of Cline (1967) replication experiment.** In this example, the looker’s head is rotated 30° to the right and the image on the bottom right depicts how this is perceived by the participant. A half-silvered mirror was used such that the looker would see only the target board through the mirror. A transparent response board was put in between the participant and the mirror such that the participant could judge at which target the looker was perceived to be looking. The black dot indicates the position of the light source.

As before, we attempted to mirror the experimental set-up reported in Cline’s study as accurately as possible. To this purpose, we built an integrated set-up (134 × 122 cm) that included the stimulus presentation mechanism as well as positions for both the looker and the participant (**Figure 5**). This provided the looker a frontal view at the target board and the participant a frontal view at the looker, achieved through a half-silvered mirror. The mirror was placed at 61 cm from both the looker and the participant, creating an apparent distance of 122 cm between each other. The target board was the same as in Cline (1967) and consisted of 13 targets located on the horizontal meridian (the only targets relevant for this experiment). These targets ranged from -12° to 12° in steps of 2° except for the -6° and 6° targets which were not included (as they were also not included in Cline’s setup). Please note that targets with a negative sign were located to the left of the midpoint. The center target (0°) was arranged such that it was aligned with a straight view of the looker and with the height of his/her eyes. The targets were composed of small light bulbs which could be lit individually by the experimenter. A half-silvered mirror was put at a 45° angle in between the position of the looker and the target board such that the participant could perceive at which target the looker was looking, yet for the looker only the target board was visible. In between the mirror and the participant, a transparent response board consisting of 17 targets was placed. The elements on the response board ranged between -16° and 16° of visual angle in steps of 2° of visual angle. To facilitate the discriminability of the response elements, each was given a distinct shape and color. Black curtains were placed such that the participant and the looker could not see each other in their peripheral vision; neither could the looker see the target board. A light source was placed in the box, on top of the mirror, oriented along the line of regard of the looker, and tilted 25° downward to be focused on the face of the looker to ensure that the lookers’ face was properly visible. A head- and chinrest was used to stabilize the head of both the participant and the looker.

#### Procedure and Design

On each trial, the experimenter (sitting behind the target board), would light up one of the target light bulbs at which the looker needed to fixate (with his/her head straight ahead or oriented 30° to the right). After this, the participant was allowed to open his/her eyes and had to judge at which target the looker was looking by naming a shape and its associated color to the experimenter. After taking note of the response, the experimenter proceeded to the next trial at the start of which the participant again had to close his/her eyes. Each target (-10°, -4°, 0°, 4°, and 10°) was fixated 20 times in each head orientation condition (straight ahead or 30° to the right). To avoid response sets, filler trials were included in each block of trials (-12°, -8°, -2°, 2°, 8°, 12°). A block of trials consisted of five repetitions of each target stimulus and one filler target each, yielding a total of 31 trials per block. Each head orientation condition was tested in a block-wise fashion, alternating between blocks for eight blocks in total (four blocks per head orientation), yielding a total of 248 trials. The order of blocks was counterbalanced across participants. Within each block, the order of trials was completely randomized. A 30° head rotation to the right was achieved by rotating the chinrest 30° as well as letting the looker fixate at a point located at a 30° angle each time before fixating a target.

### Results and Discussion

**FIGURE 6:**
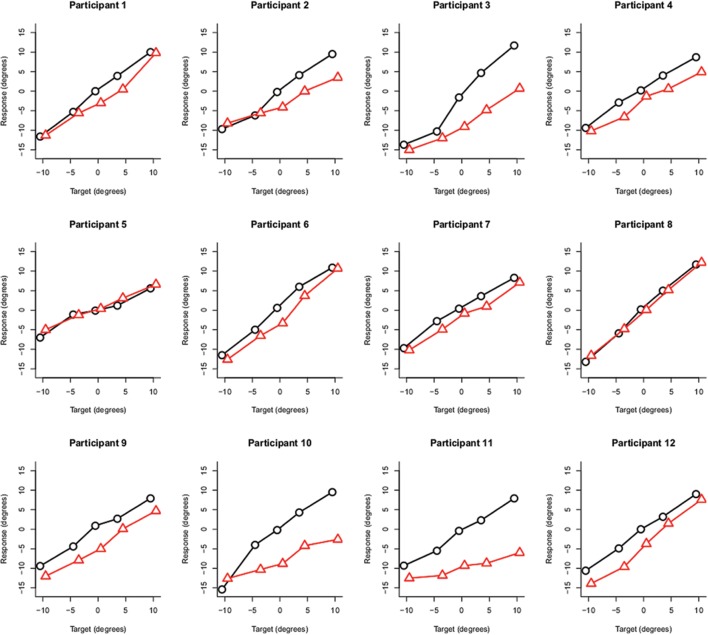
**Individual data.** For each participant, the estimates of the mean of each fitted distribution are plotted in function of each combination of target and head orientation (black = head straight; red = head oriented 30° to the right).

**FIGURE 7:**
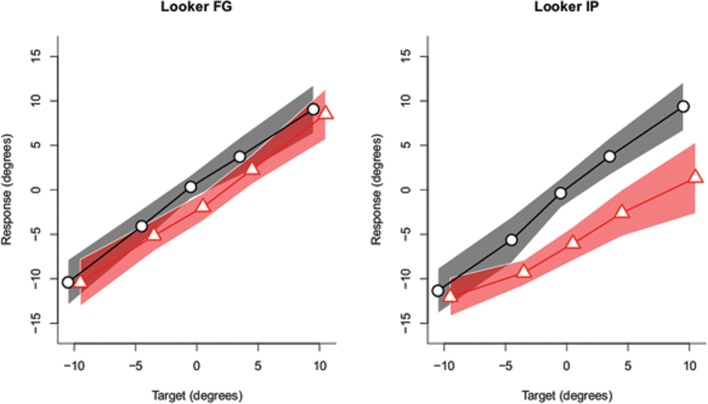
**Aggregate data.** For each combination of head orientation (black = head straight; red = head oriented 30° to the right) and target, the estimates of the mean of the fitted distribution are plotted. The shaded area denotes the 95% within-subjects confidence interval of the means of the fitted distributions. Please note that an overshoot effect amounts to a shift downward of the red line compared to the black line. The points plotted are slightly jittered to the left (black) and to the right (red) to avoid overlap when plotted.

After the experiment, the participants’ responses were recoded by the experimenter into degrees of visual angle. The filler trials were not considered in the analysis. The same strategy as in the previous experiment was applied to analyze the data. That is, for each participant, we fitted Gaussian distributions to the frequency histograms of target responses for each combination of target and head orientation. This yielded ten estimates per participant for the mean and standard deviation. **Figure 6** depicts the estimates of the mean of each fitted distribution for each participant separately. The aggregate data are presented in **Figure 7.** In this experiment, an overshoot effect would imply that, for a head oriented 30° to the right, the estimates of the mean are shifted to the lower end of the response range (i.e., more toward the left). As is apparent from **Figure 6** and in line with the results from the other replication experiment, there was considerable interindividual variability present in the data. Two participants did not show an effect of head orientation at all (participants 5 and 8). The pattern predicted by an overshoot effect (partially) held true for the remaining 10 participants and is more clearly discernible in the aggregate data depicted in **Figure 7.** An initial analysis of the data indicated that the two best fitting models included interaction effects between the looker and either the effect of head orientation or target. Therefore we decided to split up the analysis by looker and these results are reported in **Table 2.**

As is apparent, for both lookers the best models included an effect of head orientation, yet for looker FG the evidence for an effect of head orientation was not convincing according to the BF analysis. Nevertheless, for both lookers, the data both went into the direction predicted by an overshoot effect. For looker IP, the best fitting model also included an interaction between target and head orientation on top of two main effects. This interaction indicates that when the lookers’ head was oriented to the right, this not only shifted the data downward, yet also led to a shallower slope as is apparent from **Figure 7** (right).

## Discussion and Conclusion

In this paper, we critically discussed two of the three pioneering papers published in the 60s concerning the influence of head orientation on perceived gaze direction. We highlighted that they have been interpreted differently throughout the years and the goal of our discussion was to critically examine which interpretation is most likely when taking both papers at face value. In addition, we sought to replicate the experiment reported in Gibson and Pick (1963) as well as Experiment IIB reported in Cline (1967).

In our discussion of both studies we highlighted that, to our surprise, the study of Gibson and Pick (1963) actually was the more ambiguous of the two papers and cannot be unequivocally interpreted as showing either an overshoot or towing effect. With respect to the results of Cline’s (1967) Experiment IIB, however, we highlighted that an important part of the set-up of Cline’s experiments had not been considered up to now. That is, to present the looker’s gaze to the subject, Cline used a mirror. Due to this, a looker gazing to his/her right, is also gazing to the right of the subject. This implies, as we discussed, that there are no ambiguities as to whether a head oriented to the left or right should be interpreted from the viewpoint of the looker or the participant. Thus, the results more than likely can be interpreted as a towing effect.

To shed further light on this issue, we decided to run a replication of both the experiment reported in Gibson and Pick (1963) as well as Experiment IIB reported in Cline (1967). In the case of the Gibson and Pick (1963) study, our results clearly indicated an overshoot effect in nearly every participant. In the case of the replication of the Cline experiment, the results were a bit more nuanced. In general, evidence was found for an overshoot effect when the head was rotated 30° to the right, yet this effect was attenuated for one of our lookers. However, in none of our participants, we observed a towing effect. In sum, our results indicate that perceived gaze direction is biased in the direction opposite to the head turn both in a dyadic as well as a triadic gaze task.

As highlighted in the introduction, two different biases in perceived gaze direction have been reported in the literature, overshoot and towing. How do our findings relate to the bulk of findings related to the perception of gaze direction in studies following up on the papers published in the sixties? On the face of it, of the few studies that actually considered biases in gaze perception almost every study has obtained evidence for an overshoot effect (Noll, 1976; Masame, 1990; Todorović, 2006, 2009). Indeed, there is only a small set of studies that have reported on a towing effect (Maruyama and Endo, 1983; Langton et al., 2004). Thus, the fact that we obtained a data pattern consistent with an overshoot effect is in agreement with the majority of the findings on biases on perceived gaze direction.

An aspect that was particularly revealing in our results, and which has been much less appreciated in the early studies was the considerable interindividual variability present in the data set. That is, some observers appear to be less subject to biases in perceived gaze direction (as derived from the tasks used in the experiments) or differ in the width of the tuning curves through which we summarized the data. As only averaged data were reported in the original studies, it is difficult to judge how comparable the tuning curves for our data sets are to those obtained in the 60s. The cause of this interindividual variation remains largely unknown, however. One aspect that could significantly contribute to task performance is the particular looker used in the experiment. Because we manipulated the looker in a between-subject manner, it is unfortunately impossible to determine whether individual variation in perceived gaze direction is due to the influence of the lookers or to genuine interindividual variability in the mechanisms through which perceived gaze direction operates. Furthermore, interindividual differences could reflect a differential response strategy used by participants. As participants were given unlimited response time, some might have not relied on their first gaze impression to respond. Nevertheless, it should be noted that recent studies have indicated that observers with autism spectrum disorder or schizophrenia show remarkable differences in attentional orienting to gaze stimuli (Ashwin et al., 2015; Langdon et al., 2016). Furthermore, the so-called “cone of gaze” –a measure of the range of gaze angles of which an observer judges he/she is being looked at– has been observed to be wider in observers with social phobia (Gamer et al., 2011; Jun et al., 2013). It is not completely clear, however, whether this also indicates that perception of gaze direction *per se* is deficient in these populations (Pell et al., 2016). A particularly interesting avenue for future research would therefore be to further explore the consistency and extent of interindividual variability on tasks such as those used in this study, because they provide reliable psychophysical measurements. This could either be done by considering interindividual variability in the typically developing population, or by taking an extreme group approach. Although these reflections on inter-individual differences certainly provide avenues for future research, they also reveal one of the most important limitations of the current study. That is, the sample size (*N* = 12) used in both replication experiments is fairly low. Despite the fact that sample sizes such as the one used here are fairly common in both early and more recent studies on gaze perception, it inherently limits the extent to which one can arrive at strong conclusions regarding a potential overshoot or towing effect if not every single participant consistently shows an effect. With respect to the current study, we think that the data are most consistent with a bias in perceived gaze direction in the direction of an overshoot effect, given that the direction of the bias never reversed for any participant in our experiments. Experiments relying on a live looker are quite times consuming, and it is inherently more difficult to arrive at substantial sample sizes using this kind of paradigm. Nevertheless, future studies should try to sufficiently increase their sample size such that the robustness of biases in perceived gaze direction can be more thoroughly studied. Furthermore, increasing sample size provides an ideal basis to consider the natural variability present in biases in perceived gaze direction.

Although our immediate goal for this study was not to resolve any discrepancy between the studies performed in the 60s, our own observations do inevitably prompt the question as to why Cline observed a towing effect in a situation in which, like in Anstis et al. (1969) or ours, generally an overshoot effect has been reported. We outline three possibilities that might have influenced the results of Cline (1967) and which could provide guidance for future research. First, Otsuka et al. (2014) remarked that Cline used several light sources in his experiment. A study by Troje and Siebeck (1998) highlighted that perceived orientation of human heads could be influenced by the direction at which they are illuminated in the direction opposite to the illumination. Whereas Cline used light sources located to the right of the looker, we used light sources that were located in front of the looker. Nevertheless, it is not immediately clear how this difference in lighting would explain a reversal of the effects. That is, a light source located to the right of the looker would be perceived as being located on the right side of the looker’s face from the participant’s viewpoint. This would induce an apparent shift of the head orientation to the left. However, all biases reported in Cline are ones in which the error is to the right of the target. Thus, if the source of illumination would have had a particularly compelling effect, a more pronounced overshoot effect (rather than a towing effect) would have been observed given that perceived head orientation is already biased in the direction opposite to the head turn given the side of the face that was illuminated.

Second, recent studies have implicated body orientation as a potentially important and informative cue for perceiving gaze direction (Hietanen, 2002; Seyama and Nagayama, 2005; Pomianowska et al., 2012; Ashwin et al., 2015; Cooney et al., 2015; Moors et al., 2015). Thus, in these early studies, body orientation might not have been controlled in the same way across studies. Indeed, some authors might have rotated body orientation together with head orientation keeping both orientations congruent whereas others might have fixed body orientation to 0° rendering it incongruent with the head when it is rotated to the left or right (as we did in our experiments).

Third, we observed a pronounced effect of the looker in the replication of Cline’s Experiment II. That is, in one of our lookers the overshoot effect was considerably attenuated and did not reach significance anymore (although its direction was not reversed). Thus, looker-related biases might also have influenced the observations of Cline; although it is unlikely they caused a complete reversal of the effect.

To conclude, we conducted two replications experiments of studies performed in the sixties, and observed overshoot effects in both experiments which appear to be consistent with the majority of studies on the effect of head orientation on perceived gaze direction in dyadic and triadic gaze tasks.

## Author Contributions

PM and FG conceived of the experiments. TD, IP, and FG collected the data. PM analyzed the data and drafted the manuscript. All authors provided critical revisions and approved the final version of the manuscript.

## Conflict of Interest Statement

The authors declare that the research was conducted in the absence of any commercial or financial relationships that could be construed as a potential conflict of interest.

## Footnotes

1 Note that this discussion of Gibson and Pick’s (1963) results is based on the accepted interpretation that they observed an overshoot effect.

2 Please note that this low number of selected papers is due to the fact that very few of the citations explicitly interpret the results of either study.
