# Supplementary material for: The Effect of Head Orientation on Perceived Gaze Direction: Revisiting Gibson and Pick (1963) and Cline (1967)
Source: Front Psychol. 2016 Aug 10;7:1191. doi: 10.3389/fpsyg.2016.01191 (PMC4979522; doi:10.3389/fpsyg.2016.01191)
Supplement: Supplementary file 1 [file Data_Sheet_1.DOCX]

***Supplementary Material***

**The effect of head orientation on perceived gaze direction: Revisiting Gibson and Pick (1963) and Cline (1967)**

Pieter Moors^1*^, Karl Verfaillie^1^, Thalia Daems^1^, Iwona Pomianowska^2^, Filip Germeys^1,3^

^1^Laboratory of Experimental Psychology, Department of Brain & Cognition, University of Leuven (KU Leuven), Leuven, Belgium.

^2^The Leon Schiller National Higher School of Film, Television and Theatre, Lodz, Poland

^3^Department of Work and Organization Studies, University of Leuven (KU Leuven), Leuven, Belgium

* Correspondence: Pieter Moors: [pieter.moors@ppw.kuleuven.be](mailto:pieter.moors@ppw.kuleuven.be)

1. **An extended discussion of the findings of Gibson and Pick (1963) and Cline (1967)**
   1. **Gibson and Pick (1963)**

The study of Gibson and Pick (1963) aimed at documenting the accuracy of perceived gaze direction for different eye and head directions. The authors make the important remark that the perception of gaze direction is not based on “the eye-form as such but the eye-form relative to the face-form” (p. 389) – note that this observation is made independently from Wollaston (1824), as they do not cite it. The accuracy of perceived gaze direction was tested by combining three different head orientations (30° left, 0°, 30° right) with seven different eye target positions. The participant’s task was to report for each combination whether the looker was looking directly at him/her or not (yes/no) which is known as a dyadic task.

The standard deviation of the distribution of “yes” responses for each target position was taken as a measure of precision of the judgments. Since they were not very different for the different head orientations, Gibson and Pick concluded “that acuity of perception of gaze is independent of head pointing” (p. 391). Although the precision of judgments did not differ between head orientations, the distributions of “yes” responses were shifted for heads oriented to the left and right. Specifically, these distributions were shifted “in the direction of head pointing” (p. 391).

However, throughout their paper Gibson and Pick never explicitly define whether “head to left” and “head to right” of the “looker” should be interpreted from the looker’s or the subject’s viewpoint. Therefore, a shift “in the direction of head pointing” does not unambiguously refer to an overshoot or towing effect. Figure 1 depicts the results from their Figure 4 and shows that, depending on the frame of reference in which the head orientation is defined, either an overshoot or towing effect is observed.


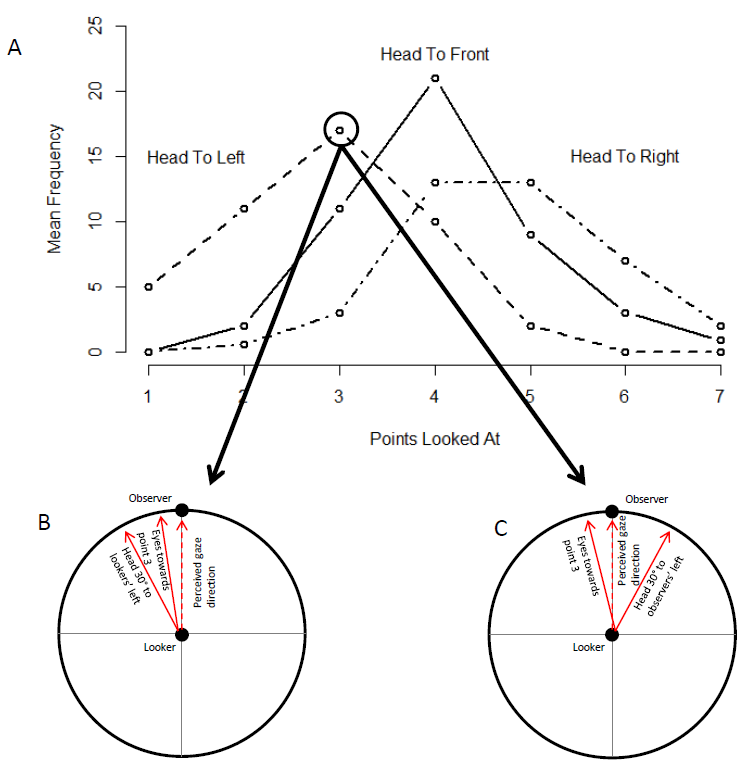


*Figure 1.* Panel A depicts the results of Gibson and Pick (1963) as they reported in their Figure 4. Panel B shows how the highlighted data point in panel A can be interpreted as the result of overshoot. In this case, head to left of the looker is defined from the looker’s perspective. Panel C on the other hand shows how this data point can be interpreted as the result of towing. In this case, head to left of the looker is defined from the subject’s perspective.

To exemplify this ambiguity we will take the data point that has been highlighted in panel A of Figure 1. Here, the looker was looking at point 3 with her head rotated to the left. The dependent variable indicates the mean frequency of responses on which the subjects indicated that the looker indeed was looking at him/her. Panel B depicts the situation in which this data point can be interpreted as the result of an overshoot effect. In this case, “head to left” is defined from the looker’s perspective. The looker’s eyes are oriented towards point 3 on the target axis and this situation yields the highest frequency of “looking at me” responses. This means that a head oriented to the looker’s left and looking at a target to the left of the subject (from the looker’s point of view) elicited *more* “yes” responses than the same head orientation with the looker gazing directly at the subject. Thus, the gaze direction of the looker is perceived to be somewhat further in the direction opposite to the direction of her head which amounts to an overshoot effect. Panel C, on the other hand, depicts the situation in which this same data point can be interpreted as a towing effect. Now, “head to left” is defined from the subject’s viewpoint and the looker is still looking at point 3 on the target axis. In this situation, perceived gaze direction actually lies in between the orientation of the eyes and the head which conforms with a towing effect. Thus, solely from the description of the methods and results we cannot make an unambiguous conclusion about what the results obtained by Gibson and Pick (1963) actually indicate. This stands in stark contrast with the prevailing assumption that an overshoot effect was observed in this study.

This prompts the question as to whether there are any indications elsewhere in Gibson and Pick’s article that would allow us to make a conclusion on which effect has been observed in this study. First, we consider the legend of their Figure 2. This figure exemplifies their statement that perceived gaze direction is not solely based on the orientation of the eyes but influenced by head orientation. The direction of the eyes and gaze is described here from the readers’ and thus the subject’s viewpoint! A further indication that left and right possibly could be interpreted from the subject’s perspective is given on p. 390: “… she [i.e., the looker] would adopt one of seven eye-postures in one of three head postures relative to the O[bserver].” Head postures relative to the subject could indicate that these should be interpreted from the subject’s viewpoint. If we would extend this to the results of the study, this would imply an interpretation consistent with a towing effect. Second, Gibson and Pick (1963) introduce the effect of head orientation on perceived gaze direction by using the Wollaston illusion, an example of the towing effect. Throughout their discussion of their results, Gibson and Pick never highlight that their results stand in contrast with this Figure. Therefore, one might reason that this indicates that Gibson and Pick interpreted their results as an overshoot effect. Nevertheless, we reason that Gibson & Pick used this Figure as an example of how head orientation can influence perceived gaze direction, rather than wanting to link it to their experimental set-up. In the case of an overshoot effect, their Figure 2 does not necessarily stand in complete opposition to their results. That is, in the literature, towing is often used in two different ways. As described in Todorovic (2006), towing as indicated by the Wollaston illusion is actually a trivial implication of the way in which our body is constructed. If my eyes are rotated 15 degrees to the left relative to my head, and I want to keep this relative angle while turning my head, the point at which I will look in space also will change. Hence, perceived gaze direction also changes. However, towing can also happen when a looker looks at the same point in space and perceived gaze direction changes in function of head orientation, in the direction of head pointing. This refers to a situation similar for overshoot, in the sense that the gazed-at location remains fixed, yet the direction in which perceived gaze direction is biased is opposite to the one that would be predicted by an overshoot effect. It is the latter kind of towing we use throughout the paper, and therefore we think the example Gibson & Pick have used in their paper also does not have any implications for the results they observed (i.e., that the results would be in contradiction with their Figure).

On the other hand, considering the experimental set-up and the results in Figure 1 panel A, together these could indicate an overshoot effect. That is, by rotating their Figure 3 90° counterclockwise, the target axis is the same as the x-axis of Figure 1 panel A. If the x-axis of Figure 1 panel A should be read with the subject and looker “in mind” underneath it, then it would make most sense – given how the looker and the subject are depicted in their Figure 3 – that “head to left/right” is defined from the looker’s perspective which would amount to an overshoot effect in the results. Second, the phrasing that the “shift is in the direction of head-pointing” (p. 391) at first might be taken as evidence for a towing effect. However, it should be noted that “shift” here refers to the shift of the distributions of “frequencies of yes judgments”, and not a shift in the perceived gaze direction. This is particularly important because otherwise this phrasing would indeed be consistent with a towing effect. As we will outline below, intuitively, this sentence provides evidence for the interpretation of their effect as an overshoot rather than a towing effect. However, we can only base this on a reading of the paper that intuitively makes sense, rather than provide a conclusive argument on the interpretation of the results. If “head to left” and “head to right” refer to left and right from the looker’s perspective, the highest frequency of “looking at me” responses is observed when the looker gazes at numbers 3 and 5, respectively. This implies an overshoot effect, because when the looker looks at target number 3 with her head rotated to her left, she is perceived as if she looks towards the participant. As such , the distributions of these frequencies indeed “shift in the direction of head pointing”. Intuitively, it is much harder to reconciliate this sentence with the opposite case in which a towing effect was observed. That is, if “head to left” or “head to right” imply that the looker’s head was rotated to the left or right of the participant, the highest frequency of looking at me responses was observed when the looker looked at point 5 or 3 while rotating the head to the right or left respectively. So, the shift for the perceived gaze direction here is in the direction of head pointing, but it’s much less intuitive to explain the shift of the distributions also in terms of head pointing. That is, a shift of the distributions to the right would be consistent with a head orientation to the right of the participant, implying that the looker actually points her head to her left. Taken literally, it is possible that Gibson & Pick described the results in this way, but it seems a much less intuitive way of describing the shifts of the distributions as in the “direction of head pointing”.

In sum, although the results of Gibson and Pick (1963) never triggered any discussion, we argue that, based on our discussion of their results, no unambiguous conclusion can be made as to whether these results indicate an overshoot or towing effect. The fact that the majority of papers discuss these results as an overshoot effect presumably stems from Anstis et al.’s (1969) own interpretations of these ambiguities. That is, Anstis et al. (1969) were the first to indicate that the results should be read as “an apparent shift of L[ooker]’s gaze to S[ubject]’s right, i.e., in the opposite direction to L’s head movement” (p. 477). Although we considered some indications in the text that could give rise to one of the two interpretations, neither of these appeared to be conclusive with respect to interpreting the results.

We now move on to a discussion of the results of Cline (1967). Ironically, these results have been the subject of debate ever since they were published, but according to our discussion they unambiguously point to a towing effect.

- 1. **Cline (1967)**

The study of Cline (1967) is, along with Gibson and Pick (1963) and Anstis et al. (1969), one of the three pioneering papers from the sixties investigating the influence of head orientation on perceived gaze direction. Confusion has arisen about the results reported in this paper ever since it was published. Consequently, different interpretations have been suggested (Anstis et al., 1969; Kluttz et.al, 2009; Langton, 2000; Langton et al., 2004; Vine, 1971; von Cranach & Ellgring, 1973). The main point of discussion revolves around the results of Experiment IIb. The results of this experiment are, on the one hand, interpreted as confirming the results of Gibson and Pick (1963) and thus, given the broad consensus of Gibson and Pick (1963) reporting an overshoot effect, as such. On the other hand, other authors have argued that the results Cline (1967) reported in Experiment IIb are in line with the so-called towing effect (Marumaya & Endo, 1983). In our analysis of the literature, we found that ten out of twenty-one articles interpreted these results in terms of an overshoot effect and eleven in terms of a towing effect.

The goal of Cline’s (1967) paper was to “(1) replicate these [Gibson & Pick’s, 1963] results and (2) to consider acuity for targets other than those located between S[ubject]’s eyes” (p. 42). To arrive at these objectives, Cline devised a circular target board in which several targets were positioned in a cross-hair fashion. In Experiment 1, the looker gazed at a subset of these targets – with a 0° head orientation, thus straight forward – and the observer had to indicate at which target the looker was looking. Thus, the acuity in both the horizontal and vertical direction was measured. The observed acuities were somewhat finer than those reported by Gibson and Pick (1963). In Experiment IIb, Cline intended to study the effect of a head turn on the accuracy of perceived gaze direction. He used only one head turn, 30° to the right. The vertical gaze directions were eliminated from the stimulus set. Thus, the set-up of this experiment was in close agreement with the one of Gibson and Pick (1963), except for the nature of the task. Gibson and Pick (1963) used a dyadic task whereas Cline used a triadic task. That is, in the study of Gibson and Pick observers had to indicate whether the looker was looking at him/her or not, although the looker was looking at different targets. In Cline’s experiments, however, the looker was looking at different targets and observers had to indicate at which target the looker was looking. In this case, a third object is the locus of attention of the looker which the observer has to identify. Table 1 shows the results of Cline’s second experiment, which have been a point of discussion ever since they were published.

Table 1

Cline’s (1967) Table II. Note that the results of Cline’s Experiment IIA have been dropped here.

| Target | Experiment IIB  (Head turned right) | |
| --- | --- | --- |
|  | CE | SD |
| Left 10° | -0.54 | 5.09 |
| Left 4° | 2.64 | 6.60 |
| Center | 3.00 | 5.29 |
| Right 4° | 4.78 | 4.62 |
| Right 10° | 5.26 | 5.94 |

We will first show how it has generally been argued that the results of Cline’s Experiment IIb can be interpreted as an overshoot effect before we put forward that actually a towing effect was observed.

A first argument concerns how Cline interprets his own results. That is, in his discussion Cline mentions that his results agree with those of Gibson and Pick (1963) and thus, given the broad consensus of Gibson and Pick (1963) reporting an overshoot effect, it is argued they should be interpreted as such. Indeed, on p. 50 Cline says: “(…) there is a good deal of evidence in support of the Gibson and Pick hypothesis (…)”. However, he does not seem to imply that this applies to the results of Experiment IIb because the sentence goes on with “(…) that there is a total stimulus, combining the direction of the eyes and the orientation of the head, that defines a direction of regard. As now stated, however, this hypothesis is incomplete. Head-position and eye-position interact to produce a perceived direction which falls between these two positions.” Thus, instead of saying that he observed the same results as Gibson and Pick, Cline actually refines the hypothesis of Gibson and Pick with his own results of Experiment IIb. Moreover, Cline describes the interaction between head and eye position as a towing effect!

In his conclusion, Cline states that “the Gibson and Pick hypothesis that head position contributes to the judgment of the direction in which the eyes are looking was generally supported.” This quote presumably also has been used as a validation of the argument that Cline says that his results are in line with those of Gibson and Pick. However, Cline merely says that they are *generally* in support of the hypothesis that eye and head orientation indeed interact to yield a perceived gaze direction. Moreover, since the results of Gibson and Pick (1963), as we discussed, do not unambiguously point to an overshoot effect, it could well have been that Cline actually referred to a towing effect with respect to the agreement between both studies. Indeed, this would be consistent with how he discussed his results in his discussion.

Thus, the argument that Cline claims that his results agree with those of Gibson and Pick does not appear to be valid. Indeed, when both Cline’s discussion and conclusion are considered as a whole, Cline’s words seem to be more nuanced than plainly claiming that his results agree with Gibson and Pick and Cline actually describes the interaction between head and eye position as a towing effect.

A second argument is similar to the one in the discussion of the results of Gibson and Pick (1963). That is, Cline never specifies throughout the text whether the looker’s head orientation should be interpreted from the looker’s or the subject’s viewpoint. However, as we will argue, this argument is invalid when one considers one particular aspect of Cline’s experimental set-up which has been ignored when the results of Cline’s Experiment IIb have been interpreted. Indeed, to present the looker’s gaze to the subject Cline uses a *mirror*. Because of this mirror, there is no confusion possible as to what left and right meant throughout the paper. Indeed, if the looker turned her head to her right, the subject would see the looker turning her head to his/her right. Ignoring this aspect of the experimental set-up has important implications for interpreting the results of Experiment IIb and indeed renders the text confusing at some points.

Without considering the mirror, the looker’s head orientation can be interpreted from either the looker’s or the subject’s perspective. To arrive at an interpretation of the results in terms of an overshoot effect, the looker’s head oriented to the right must be interpreted from the looker’s perspective and, consequently, the head is oriented to the subject’s left – given that the mirror is ignored. In what follows, we will discuss how this interpretation of head orientation amounts to interpreting the results in terms of an overshoot effect.

Starting from Table 1, the targets can be interpreted in function of the target board. That is, the targets on the horizontal axis are arranged from “M to E”. In his Table I, Cline reports M4 as Left 8°. So, M4 is the target 8° to the left for both the looker and the subject. Likewise, the targets in Table 1 can be interpreted as such. With respect to the constant errors, Cline indicates on p. 49 that “the constant errors tend to be to the *S’s right*”. Thus, the constant errors in Table 1 are also best interpreted from the subject’s point of view. Thus, the left and right targets in Table 1 are on the left and right side of the subject as are the constant errors where “positive [horizontal] constant errors are to the right of the target” (p. 44, legend of Table I). However, if the looker’s head is oriented to the subject’s left – the premise from which we started – and target positions are defined from the subject’s perspective, it is not possible that the target Left 10° can be the one that is of extreme discrepancy with the head orientation. This should be the case however since Cline indicates on p. 48: “(…) except for the case of extreme discrepancy between head and eye orientation. With the head turned 30° to the right and the eyes aimed at 10° to the left of the center target, there is no CE”. Indeed, the target Left 10° is the one with the smallest CE in Table 1 and this target would lie approximately in between M6 and M4. Given that the looker’s head is oriented to the subject’s left, target Left 10° is not the target of extreme discrepancy with the looker’s head orientation although it does have the smallest CE. So, interpreted in this way, the labels would have to be switched to be in accordance with what Cline says in the text (in line with what Kluttz et al., 2009 claim). After switching, target Right 10° would then be the one with the smallest CE and also the one of extreme discrepancy between head and eye orientation. Then, the interpretation of the results is straightforward. Except for the target on the far right side, every target position is overestimated to the right side of the subject because the associated CEs are all positive. Indeed, since the head is oriented to the left of the subject and the gazed-at location is estimated as further than it actually is, this provides evidence in favor of the overshoot effect. This situation is depicted in Figure 2. In this figure, Table 1 is depicted graphically with the response board as a reference frame. As is apparent from the figure, all errors (circles) lie to the right of the targets (crosses) and target Right 10° is the one with the lowest CE (smallest difference between target and error). With the looker’s head in mind oriented to the left of the subject, every target except Right 10° is overestimated.


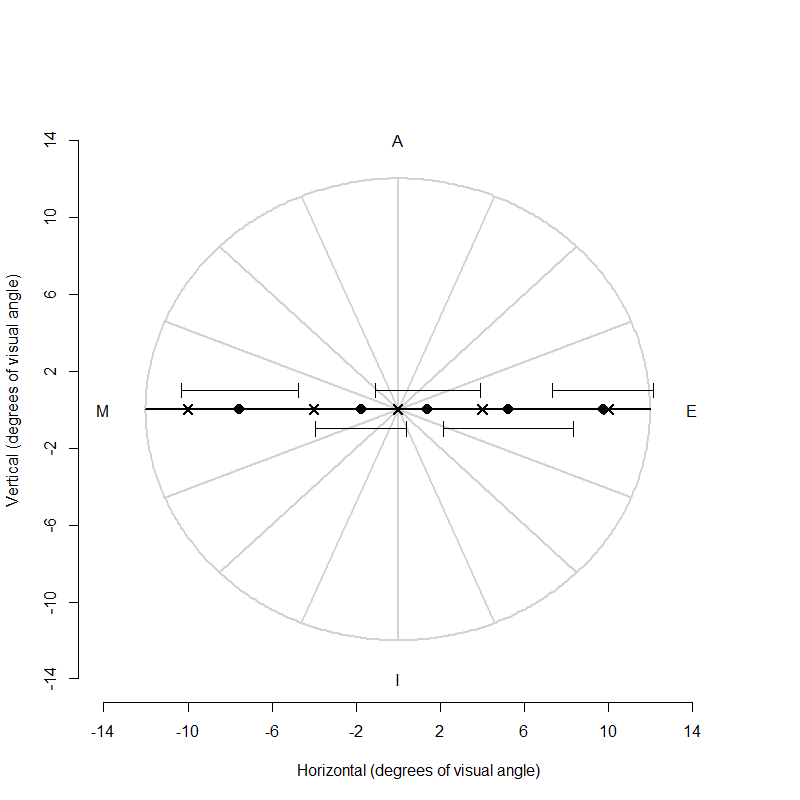


*Figure 2.* A graphical depiction of Table 1 as an overshoot effect. This figure shows the results after switching the labels in Table 1.The crosses indicate the target positions and the circles the constant errors. The error bars indicate the standard deviations for the constant errors. The axes denote the horizontal and vertical direction in degrees of angle.


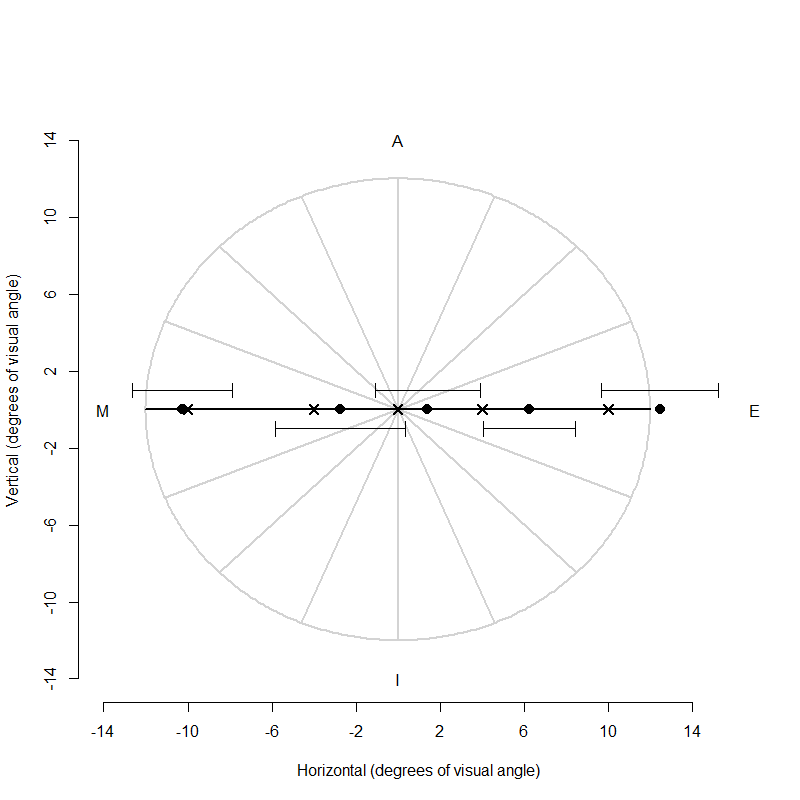


*Figure 3.* A graphical depiction of Table 1 as a towing effect. The crosses indicate the target positions and the circles the constant errors. The error bars indicate the standard deviations for the constant errors. The axes denote the horizontal and vertical direction in degrees of angle.

In sum, to arrive at the conclusion that an overshoot effect has been observed in Cline’s Experiment IIb involves ignoring the mirror in Cline’s experimental set-up and assuming that the looker’s head oriented to the right should be interpreted as to the subject’s left. Interpreting the text from this premise then needs the assumption that Cline mistakenly switched the labels in Table 1. Otherwise, the text and the labels in Table 1 would not be consistent anymore. Only after switching the labels in Table 1, the results can be interpreted as an overshoot effect.

As we already highlighted, our main argument to invalidate the argument that Cline never specified from which viewpoint left and right should be interpreted is the use of a mirror to present the looker’s gaze to the subject. This aspect of the experimental set-up implies that there are no ambiguities in the text with respect to the interpretation of left and right and the results reported in Table 1 can be interpreted as they are reported (for a graphical depiction see Figure 3). In this case, target Left 10° indeed is the one of extreme discrepancy between eye and head orientation. Furthermore, Cline’s discussion of the constant errors as being “in the direction of the turn of the head” (p. 47) and his summary of the results as “Head position and eye position interact to produce a perceived gaze direction which falls between these two positions” (p. 50) are no longer confusing since they all fit with an interpretation in terms of a towing effect.

1. **Supplementary Tables**

Table S1. An overview of the articles citing the studies of Gibson and Pick (1963) and/or Cline (1967).

| Article | Gibson and Pick (1963) citation | Cline (1967) citation | Interpretation Gibson and Pick (1963) | Interpretation Cline (1967) |
| --- | --- | --- | --- | --- |
| Anstis, Mayhew, and Morley (1969) | The CEs, unlike thresholds, were altered by the head position: turning L's head caused an apparent shift of L's gaze. To paraphrase Gibson's results: When L aimed his head at S's left shoulder but looked askance at S's left ear, then S perceived L to be looking straight at him. Gibson called this an apparent shift *in the direction* of L's head movement. We propose to call it an apparent shift of L's gaze to S's right, *i.e. in the opposite direction* to L's head movement. (p. 477) | Large CEs were reported *"in the direction* of the turn of the head." Cline did not make it absolutely clear what this means, but he remarked that his results agreed with Gibson's. So it appears that he found, as did Gibson and the present writers, that if L's head pointed toward S's left shoulder, then L's gaze at S's left ear seemed to S to be directed at S's nose. (p. 478) | O | O |
| Vine (1971) | Gibson & Pick (1963) concluded that GD judgments are likely to approach the human acuity limits in terms of discrimination, but where the head is turned away from the axis of GD, deviations of the GD itself from the sender-receiver axis will be underestimated by an amount dependent on the HD (p. 321) | Cline (…) confirmed that there is underestimation of deviations when the head is turned independently (p. 321) | O | O |
| von Cranach & Ellgring (1973) | (…) Gibson and Pick (1963), found that gaze deviation from the direction of head-turn was over-estimated rather than under-estimated (p. 442) | it appears that Cline (1967) may have found that perceived direction is intermediate between eye direction and head direction (p. 442) | O | T |
| Deutsch & Auerbach (1975) | We find, as did Gibson and Pick, constant errors of judgment of direction of the gaze in the direction of head pointing (p. 476) | *Cline (1967) is not explicitly discussed* | O | / |
| Argyle & Cook (1976) | Gibson and Pick found that turning the head 30° to one side produced a constant error of about 3° in the perceived line of regard (p. 49) | Cline likewise found that perceived direction of gaze was intermediate between the orientation of eyes and head (p. 49) | T | T |
| Noll (1976) | The results showed a surprisingly constant error of judged gaze in a direction opposite to the direction of the model's head turn. (p. 632) | The result of this experiment confirmed Gibson and Pick's discovery of a constant error with head turn in the perception of being looked at. (p. 632) | O | O |
| Marumaya & Endo (1983) | perceived direction of gaze is intermediate between the orientation of eyes and head (p. 126) | perceived direction of gaze is intermediate between the orientation of eyes and head (p. 126) | T | T |
| Masame (1990) | Anstis et al. (1969) also reported the head turn effect. Their finding that the apparent gaze direction is shifted in the opposite direction to the looker's head turn coincided with the result to Gibson & Pick (1963). (p. 35) | And probably the result of Cline (1967) shows the same effect. (p. 35-36) | O | O |
| Gale & Monk (2000) | Displacing the gazer's head by rotating the chinrest so that his or her head was facing 30° to the right or the left had only small effects, producing a slight bias in the estimates of 3° in the direction the head was facing. (p. 587) | Cline also varied head position and obtained small effects of bias that were in line with Gibson and Pick's (1963) result. (p. 587) | T | T |
| Langton (2000) | Gibson and Pick established that when the looker’s head was turned to one side, subjects tended mistakenly to judge gaze directed 2.98 to the opposite side as actually looking directly at them. They concluded that the perception of eye pointing is evidently influenced by the perception of head pointing (p. 828) | Cline (1967) refined Gibson and Pick’s (1963) conclusion, claiming that head orientation and eye gaze direction interact so that the direction of attention is perceived as falling somewhere between these two positions (p. 828) | O | T |
| Langton, Watt, and Bruce (2000) | The second kind of influence of head angle on the perception of gaze is a kind of overshoot effect. Imagine someone standing in front of you with their head 30 degrees or so to your right and with their eyes either staring straight back at you, or back towards your left shoulder. Under these conditions, it is likely that you will perceive their eyes to be gazing a little further to the left than they actually are (p.55) | (…) the perceived direction of gaze can be ‘towed’ towards the orientation of the head. In this case, as with the Wollaston images, the direction of gaze is perceived to be somewhere between the angle of the head and the true line of regard of the eyes (p. 55) | O | T |
| Wilson, Wilkinson, Lin, & Castillo (2000) | In addition, constant errors were observed such that the counter-rotated eye direction was overestimated in the direction opposite to the head orientation (p. 460) | *Cline (1967) is not explicitly interpreted* | O | / |
| Langton, Honeyman, Tessler (2004) | For example, imagine someone standing in front of you with his or her head 30º or so to your right and with his or her eyes either staring straight back at you, or back toward your left shoulder. Apparently, under these conditions, you might perceive his or her eyes to be gazing a little further to the left than they actually are (p. 754) | First, under certain circumstances, the perceived direction of gaze can be “towed” toward the orientation of the head. In this case, the direction of gaze is perceived to be somewhere between the angle of the head and the true line of regard of the eyes (p. 754) | O | T |
| Imai, Sekiguchi, Inami, Kawakami, & Tachi (2006) | He also reported that judged targets were shifted into opposite direction of the head rotation when the looker rotated her head and looked askance at targets (p. 124) | Cline reported the same shift of the judged location in the opposite direction to the looker’s head orientation (p. 124) | O | O |
| Todorović (2006) | Biases in the *opposite* direction were reported by Gibson and Pick (1963), … (p. 3550) | Biases in the *opposite* direction were reported by …, Cline (1967), … (p. 3550) | O | O |
| Gamer & Hecht (2007) | The orientation of the looker’s head attracted the perceived direction of the gaze cone toward the head direction (…) Comparable results were obtained by (…) Gibson and Pick (1963) (p. 713) | The orientation of the looker’s head attracted the perceived direction of the gaze cone toward the head direction (…) Comparable results were obtained by (…) Cline (1967) (p. 713) | O | O |
| Poppe, Rienks, & Heylen (2007) | In studies of dyadic gaze an underestimation of the gaze target for heads turned to either side has been reported (p. 974) | In studies of dyadic gaze an underestimation of the gaze target for heads turned to either side has been reported (p. 974) | T | T |
| West & van Veen (2007) | Three studies–– those by Anstis, Mayhew, and Morley (1969); Cline (1967); and Gibson and Pick (1963)––have measured the effect of a 30 deg head turn on the perceived direction of gaze. All researchers found that with intended eye contact the perceived direction of gaze was displaced in the direction that was opposite to the head turn (p. 314) | Three studies–– those by Anstis, Mayhew, and Morley (1969); Cline (1967); and Gibson and Pick (1963)––have measured the effect of a 30 deg head turn on the perceived direction of gaze. All researchers found that with intended eye contact the perceived direction of gaze was displaced in the direction that was opposite to the head turn (p. 314) | O | O |
| Kluttz Mayes, West, & Kerby (2009) | It was not until 100 years later that Gibson and Pick (1963) published the first study that experimentally measured the effect that head turn has on the perceived direction of gaze. In their study, when observers viewed a live model from 200 cm with the model's head turned 30° to the observer’s left, the observers felt that they were being directly looked at when the model was actually looking at their left ear. (p. 1979) | There is some confusion in the literature because Cline's Table 2 may have mixed up data for right and left. (…) Our above interpretation is based on Cline's claim that his results agree with Gibson and Pick (1963) (p. 1980) | O | O |
| Mareschal, Calder, Dadds, & Clifford (2013) | *Gibson and Pick (1963) are not explicitly interpreted* | (…) shows that when the head position and eye  position are incongruent, gaze is generally biased in  the direction of the head, such that its perceived direction falls between the two (p 2-3) | / | T |
| West (2013) | Studies have more consistently reported that the judged direction of  binocular central gaze from a turned head overestimates the true target location (p. 495)° | Studies have more consistently reported that the judged direction of  binocular central gaze from a turned head overestimates the true target location (p. 495) | O | O |
| Mareschal, Otsuka, & Clifford (2014) | Since then, some authors have reported repulsive effects of the head rotation (eyes appeared shifted away from the head direction, e.g., Anstis, Mayhew, & Morely, 1969; Gamer, Hecht, Seipp, & Hiller, 2011; Gibson & Pick, 1963; Mareschal et al., 2013a) (p. 1) | while others have reported attractive effects (gaze deviation appears shifted toward the head rotation; e.g., Cline, 1967; Langton, Honeyman, & Tessler,2004; Todorovic, 2006) (p. 1) | O | T |
| Otsuka, Mareschal, Calder, & Clifford (2014) | In a pioneering study, Gibson and Pick (1963) used real human faces as the stimuli for their exploratory experiment, and reported that perceived gaze direction was consistently biased opposite to the head orientation (repulsive effect) (p. 1426) | Unlike Gibson and Pick (1963) and others (Anstis et al., 1969; Gamer & Hecht, 2007; Noll, 1976), Cline (1967) reported that gaze direction was constantly biased toward the head orientation (attractive effect) when the head was rotated rightward by 30° (p. 1426) | O | T |
| Sheldon, Quint, Hecht, & Bowers (2014) | Consistent with prior studies, we found that the orientation of the virtual head affected the perception of straight-ahead gaze. Specifically, there was a shift of direct gaze toward the direction in which the head was rotated—an “attractor” effect—for both the horizontal and vertical planes. | *Cline (1967) is not explicitly interpreted* | O | / |
| Stiel, Clifford, & Mareschal (2014) | The account of Anstis et al. suggests that gaze should consistently be perceived in the direction opposite to the head turn as found by Gibson and Pick (1963) (p. 2) | However, it has also been reported that ‘‘head position and eye-position interact to produce a perceived direction which falls between these two positions’’(p. 2) | O | T |
| Otsuka, Mareschal, & Clifford (2015 | that the perceived gaze direction is slightly biased in the opposite direction to the head rotation (repulsive effect: Anstis, Mayhew, & Morley, 1969; Gamer & Hecht, 2007; Gibson & Pick, 1963; Masame, 1990; Noll, 1976). (p. 1) | *Cline (1967) is not explicitly interpreted* | O | / |

*Note.* O = Overshoot effect; T = Towing effect.
